# Recovery from Diabetic Coma

**Published:** 1908-04-11

**Authors:** 


### RECOVERY FROM DIABETIC COMA.

It is undoubtedly rare for a patient suffering from diabetes mellitus to reach the stage of coma, and yet to be saved from death for the time being. In the following case it can scarcely be doubted that the treatment adopted was the direct cause of the recovery :—

The patient was a man aged 25, who developed the symptoms of diabetes mellitus acutely, and without apparent cause. He had been quite well until three weeks before he was seen, and then suddenly began to pass large quantities of urine, to waste, and to become very weak. He could name the exact day when the trouble set in, as though he had not been diabetic one day, and had developed the disease by the next. So sudden an onset is unusual.

He came under observation in the hospital on March 11. He was obviously thin, wasted, and muscularly weak. His skin was dry and harsh, but his tongue was moist and clean. No abnormal physical signs could be detected in the cardiac, pulmonary, or alimentary system.

His urine had a specific gravity of 1040, with the typical colour and smell of a diabetic urine. It contained 33 grains per ounce of sugar; and as 118 ounces of urine were passed in the first twenty-four hours, he was passing about 3,894 grains of sugar per diem. Diacetic acid and acetone were both present in abundance. The retinæ looked natural on ophthalmoscopic examination. There was none of that golden yellow appearance of the retinal blood-vessels, such as has been observed when diabetic lipæmia exists in certain cases of threatening coma. The patient's knee-jerks could not be obtained, but there was no other evidence of peripheral neuritis. Previous to admission the patient had already been upon a restricted carbohydrate diet for a week, so this was now continued. Both appetite and thirst were extreme.

Two days later, on March 13, the man was noted to be drowsy, and his tongue, which had hitherto been moist, was now dry, red and shiny—perhaps because he had begun to breathe through his mouth as in air hunger. Next day he complained of a gnawing epigastric pain. This is always a very dangerous sign in one who is the subject of diabetes mellitus. It happens again and again that the first notice of imminent coma is given by what the patients themselves often describe as a "bilious attack." The man, who had hitherto eaten his food voraciously, now refused to eat anything, and he began to be very distinctly drowsy. The quantity of urine fell from above a hundred ounces down to 42 ounces in the twenty-four hours. The bowels had not been opened for two days, so four grains of calomel were given by the mouth, followed later by a soap and water enema, which produced a very good evacuation.

The drowsiness, however, steadily increased, the patient first ceasing to take any notice of things going on near him, and shortly afterwards becoming so deeply comatose that he could not be roused by any ordinary means. The breathing was at the same time typical of air-hunger, the long, deep sighing respirations following one another at slow regular intervals. There was no particularly ethereal odour in the breath, but the general condition of the patient was that of typical diabetic coma.

From the time that this coma threatened, active steps to combat it had been adopted. Sodium bicarbonate was administered both orally and per rectum in heroic doses. By the mouth it was given as a drink, of the strength of one drachm to the pint of water, and the patient was supplied with as much of this as he could be got to swallow. The rectal injections were given slowly through a funnel and long, narrow rubber tube, in a similar way to that in which large nutrient enemata are administered, the solution used being one ounce of sodium bicarbonate to each pint of water, at body temperature. Two pints of this solution were given each time, and repeated every four hours. The whole of it was retained, so that in twelve hours the patient had had six ounces of sodium bicarbonate by the bowel, and nearly an ounce more by the mouth. None was given hypodermically by infusion.

Next day, on March 15, the patient had emerged from his coma to such an extent that he ate solid food when it was placed in his mouth. He was still very drowsy, but he was sufficiently conscious to be able to tell onlookers that the dull pain in his epigastrium had almost gone. The rectal injections were continued for four days, and the alkali by the mouth was administered to the extent of two pints of the solution daily for a week longer. The coma had now long since disappeared, and the man was gaining weight. He left the hospital, still with sugar, diacetic acid and acetone in his urine, but greatly relieved for the time being.

Diabetic coma never comes on unless there is either diacetic acid or acetone, or both, in the urine; and it is generally held that these substances, or others allied to and associated with them, are the immediate cause of the coma. The latter is due, apparently, to a poisoning by acids—an "acidosis," as it is called—which result from abnormal metabolism in diabetic patients, and it is to neutralise these acids that the large doses of sodium bicarbonate are given. Of course, if the patient's tissues continue to manufacture the acid products with unabated vigour, their neutralisation by alkali can effect but very temporary relief. If, on the other hand, there is an abatement in the acidosis, the administration of the alkali may just serve, as in the above instance, to tide the patient over a period of extreme danger.

It is wonderful how much of a drug can be borne by a patient who is in need of it. A normal person would probably have exhibited acute symptoms of sodium bicarbonate poisoning if he had received as much of it in a given time as this patient did. Vomiting would almost certainly have occurred, and the rectum and colon would soon have refused to retain the injections. Neither vomiting nor diarrhœa occurred in this patient, however. Alkali was required, and comparatively huge quantities of it were utilised.

The case teaches that whenever a diabetic patient is threatened with coma, sodium bicarbonate is the drug to give, and that in urgent cases the rectum is a very valuable channel for its administration.

